# Hydrolysis of phytate to its lower esters can influence the growth performance and nutrient utilization of broilers with regular or super doses of phytase

**DOI:** 10.3382/ps/pex012

**Published:** 2017-02-16

**Authors:** L. A Beeson, C. L. Walk, M. R. Bedford, O. A. Olukosi

**Affiliations:** * Monogastric Science Research Centre, Scotland's Rural College, Edinburgh, EH9 3JG, UK; † University of Glasgow, Glasgow, G12 8QQ, UK; ‡ AB Vista, Woodstock Court, Blenheim Road, Marlborough Business Park, Marlborough, Wiltshire, SN8 4AN, UK

**Keywords:** phytase, super-doses, growth performance, nutrient utilization, phytate hydrolysis

## Abstract

The aim of the study was to observe the effects of dietary available phosphorus (aP) and calcium (Ca), with regular or super doses of phytase, on phytate hydrolysis and subsequent influences on broiler growth performance and nutrient utilization. In a 2 × 3 factorial design, 384 Ross-308 broilers were allocated to one of 6 dietary treatments with 8 replicates in a randomized complete block design for 21 days. Diets were nutritionally adequate (positive control, PC) or marginally deficient in aP and Ca (negative control, NC), with 0, 500 or 1,500 FTU/kg phytase. Bird and feed weights were recorded on d 0 and 21, excreta were collected on d 19 and 20, and gizzard and ileal contents were collected on d 21. Body weight gain (*P* < 0.01) increased linearly with phytase in the PC and quadratically in the NC. There was an interactive effect on ileal DM, N, and P utilization, increasing quadratically with phytase supplementation in the NC, but there was no phytase influence in the PC (*P* < 0.05). Phytase linearly increased copper (*P* < 0.001) and linearly decreased Ca (*P* < 0.05) utilization in the ileum. Phytase decreased ileal (IPx, inositol x-phosphate) IP6 and IP5 and increased inositol (quadratic, *P* < 0.001) but had no effect on IP4 or IP3. The influence of the dietary aP was more apparent on the hydrolysis of phytate and phytate esters after the ileum, with increasing (linear and quadratic, *P* < 0.05) IP4 and IP3 content in the excreta of birds fed the NC or PC when phytase was added. Phytate hydrolysis improves the growth potential of birds fed NC diets, allowing them to match the growth performance of birds fed PC diets and improve nutrient utilization. These results indicate that dietary Ca and aP concentrations can be reduced when phytase is supplemented. It also may be beneficial to apply the enzyme nutrient matrix to other nutrients in the diet to maintain an optimal balance of nutrients in the digesta.

## INTRODUCTION

It is well documented that non-ruminant species, particularly poultry and pigs, are unable to sufficiently utilize phosphorus (**P**) from plant-based feed ingredients due to its binding with phytate, and the animals’ inability to produce sufficient endogenous phytase. Exogenous phytase has been supplemented to commercial poultry and pig diets for many years, with the aim of improving growth performance in P deficient diets (Biehl et al., 1995; DeLaune et al., 2004), as well as reducing environmental P pollution (Angel et al., 2002). Regular doses of phytase have been reported to improve body weight gain, efficiency of feed conversion, nutrient utilization, and bone mineralization in broilers fed diets containing low available P (**aP**; Leske and Coon, 1999; Olukosi and Adeola, 2008; Selle et al., 2012). Super-dosing is the addition of phytase at levels around or above 2,500 FTU/kg (Adeola and Cowieson, 2011); however 1,500 FTU/kg is often considered a super-dose as it is much higher than the regular doses used commercially of 500 to 1,500 FTU/kg. Improvements in growth performance observed are likely to be partly a result of an increase in availability of the nutrients that had previously been bound with phytate, including protein, and minerals such as calcium, zinc, and sodium (Maenz, 2001; Ravindran et al., 2001; Applegate et al., 2003; Adeola and Cowieson, 2011). The “extra-phosphoric effects” of super-doses of phytase are benefits resulting from more than just an increase in P availability and may be a result of changes occurring within the gut and the overall health of the animal. Increased phytate hydrolysis may improve overall performance and efficiency of nutrient utilization, thereby increasing the efficiency of production for poultry farmers. It is the aim of the current experiment to consider the effect of the inositol phosphate (**IP**) esters produced during phytate hydrolysis on the growth performance and nutrient utilization of broilers fed diets containing different levels of aP, calcium, and phytase.

## MATERIALS AND METHODS

### Animals, Diets, and Housing

A total of 384 one-day-old Ross 308 broilers was used for the study, and the birds were housed in modified raised-floor pens with water and experimental diets provided on an ad libitum basis for 21 days. There were 48 cages each containing 8 birds. Birds and feed were weighed on d zero and 21 for determination of growth performance.

Birds were monitored at least twice daily, ensuring good health and that feed and water supplies were clean and adequate, and were wing-tagged at d 8 for individual identification. Diets were in mash form and fed ad libitum throughout. The house temperature was as detailed in the Ross broiler manual (Aviagen, 2014), where ambient temperature (measured at chick height) was 30°C, litter temperature 28 to 30 °C, and humidity at 60 to 70%. At d 3, the temperature was decreased at a rate of one °C per d, so that on d 21, the temperature was 22 °C. For the first 7 d, the lighting regime was set to 23:1 light: dark h, with 30 to 40 lux intensity. All procedures were approved by the SRUC Animal Experiment Committee prior to commencement.

On arrival, birds were randomly allocated to 6 treatments with 8 replicates each in a randomized complete block design and a 2 × 3 factorial arrangement. The factors included 2 levels of dietary aP (0.50% and 0.35%) and 3 levels of phytase supplementation (0, 500 FTU/kg, and 1,500 FTU/kg; Quantum Blue, E. coli phytase, AB Vista, Marlborough, UK). The positive control (**PC)** diet was formulated to meet Ross 308 energy and nutrient requirements (Ross 308 Specifications, 2007). The negative control diet was formulated to be deficient from the PC by 0.15% aP and 0.16% calcium (**Ca**), in accordance to the enzymes nutritional matrix specifications for 500 FTU/kg inclusion rate. Therefore, the dietary treatments were: (1) PC; (2) PC + 500 FTU/kg phytase; (3) PC + 1,500 FTU/kg phytase; (4) Negative control, **NC**; (5) NC + 500 FTU/kg phytase; and (6) NC + 1,500 FTU/kg phytase. Titanium dioxide was used as an indigestible marker for digestibility calculations. Table 1 shows the ingredient composition of the diets.

**Table 1.** Ingredient composition of the experimental diets.

Description of diets	PC^1^	PC500	PC1,500	NC^2^	NC500	NC1,500
Phytase, FTU/kg	0	500	1,500	0	500	1,500
Ingredients, g/kg						
Corn	346.5	346.5	346.5	382.9	382.9	382.9
Wheat	200	200	200	200	200	200
Soybean meal	302	302	302	295	295	295
Soybean oil	50	50	50	28	28	28
Di-calcium phosphate^3^	21.0	21.0	21.0	12.6	12.6	12.6
Limestone^4^	9.7	9.7	9.7	11.3	11.3	11.3
Titanium-dioxide premix^5^	25.0	25.0	25.0	25.0	25.0	25.0
CGM for enzyme^6^	30.0	20.0	0.0	30.0	20.0	0.0
Enzyme premix^7^	0.0	10.0	30.0	0.0	10.0	30.0
Vitamin-mineral premix^8^	5.0	5.0	5.0	5.0	5.0	5.0
Methionine	1.9	1.9	1.9	1.9	1.9	1.9
Lysine	3.6	3.6	3.6	3.8	3.8	3.8
Threonine	0.7	0.7	0.7	0.4	0.4	0.4
Salt NaCl	3.1	3.1	3.1	3.1	3.1	3.1
NaHCO_3_	1.5	1.5	1.5	1.0	1.0	1.0
Total	1,000	1,000	1,000	1,000	1,000	1,000

^1^PC: Positive control.

^2^NC: Negative control.

^3^Di-calcium phosphate, 25.7% Ca, 17.5% P.

^4^Limestone, 38.9% Ca.

^5^3.5 kg TiO_2_ mixed with 13.8 kg corn gluten meal.

^6^Corn gluten meal forms the basis of the enzyme premix and is used as a filler in the place of phytase.

^7^Phytase premix is mixed with corn and has an activity of 150 FTU/g.

^8^Premix supplies the following per kg diet: Vit. A, 5484 IU; Vit. D3, 2643 ICU; Vit E, 11 IU; Menadione sodium bisulfite,4.38 mg; Riboflavin, 5.49 mg; d-pantothenic acid, 11 mg; Niacin, 44.1 mg; Choline chloride, 771 mg; Vit B12, 13.2 μg; Biotin, 55.2 ug; Thiamine mononitrate, 2.2 mg; Folic acid, 990 μg; Pyridoxine hydrochloride, 3.3 mg; I, 1.11 mg; Mn, 66.06 mg; Cu, 4.44 mg; Fe, 44.1 mg; Zn, 44.1 mg; Se, 300 μg.

### Sample Collection

Birds and feed were weighed on d 0 and 21 for determination of growth performance. On d 21, 6 birds per pen (the 2 remaining birds, with body weights close to the pen average, were used for another study) were euthanized by cervical dislocation and used for collection of the entire content of the gizzard and ileum. Excreta were collected from each cage on d 19 and 20 to determine total tract nutrient retention. Ileal digesta were collected from the terminal ileum, by flushing, for determination of ileal nutrient utilization. Ileal and gizzard contents were analyzed for phytate P and inositol esters of phytate.

### Processing of Samples

#### Chemical Analyses of the Diets and Digesta.

Diets and digesta were dried in a forced draft oven at 80 °C, for a minimum of 48 h or until a constant dry weight was reached, and ground through a 0.5 mm sieve. Nitrogen, DM, minerals, and gross energy were analyzed using AOAC procedures (955.04, 968.04). Inositol phosphate esters and inositol were determined by high-performance ion chromatography-based techniques, similar to those used by Blaabjerg et al. (2010), refined by the University of East Anglia. Phytate-P was predicted by NIR (ESC Standard Analytical Method, SAM120; AB Vista). Non-phytate P was calculated by subtracting phytate-P from total P. Phytase was analyzed by ELISA specific for Quantum Blue (ESC Standard Analytical Method, SAM099; AB Vista), in a method similar to that described by Engelen et al. (2001). One unit of phytase is defined as “the quantity of enzyme that will liberate 1 mol inorganic ortho-phosphate per minute under the conditions of the assay” (Engelen et al., 2001).

#### Titanium Digestion.

Determination of titanium concentration in diet, excreta, and digesta samples were performed as described by Short et al. (1996). Each sample was analyzed in duplicate and the absorbance measured using the Jenway 6300 spectrophotometer (63-Zero, software version 1.2.3008.30151), at an absorbance of 410 nm. The determined values were then used for calculation of utilization of the various nutrients.

### Calculations and Statistical Analysis

Feed disappearance and BW were recorded to calculate feed intake (**FI**) and BW gain (**BWG**). Feed conversion ratio (**FCR**) was calculated from the BWG and FI data. Feed intake was corrected for mortality through calculation of bird-days. Statistical analysis of mortality % was performed following the application of 1/(sqrtX+0.1) to allow for analysis of zero mortality.

#### Nutrients.

In the ileum, nutrient digestibility reflects the nutrients utilized, whereas in the excreta this reflects the overall nutrients retained from the diet. Nutrient digestibility was calculated using the following equation:

\begin{eqnarray*}
&&{\rm Nutrient\ digestiblity\ (\%)}\\
&&\quad = \left[ 1 - \left( {\frac{\rm Ti\ in\ diet} {\rm Ti\ in\ sample}} \right)
\times \left( {\frac{\rm Nutrient\ in\ sample} {\rm Nutrient\ in\ diet}} \right) \right]\nonumber\\
&&\qquad \times \, 100
\end{eqnarray*}

Dry matter digestibility was calculated by:

\[
{\rm Dry\ matter\ digestibility\ (\%)} = 1 - \left( {\frac{\rm Ti\ in\ diet}{\rm Ti\ in\ excreta}} \right)
\]

Means (for each pen) were pooled for each of the diets to allow comparison of the treatment effects. Statistical analysis of the data was done using the General ANOVA function of Genstat (14th Edition, VSN International Ltd.). Data were checked for normality and suitably transformed when required and analyzed as a 2 × 3 factorial where the model included aP level, phytase, and the interaction. Statistical significance was considered at *P* ≤ 0.05. When the main effect of phytase was significant, orthogonal polynomial contrasts were used to assess linear and quadratic treatment effects. Contrasts were adjusted for unequal spacing of phytase doses, with the linear contrast being −0.61, −0.15, and 0.76, and the quadratic contrast being 0.53, −0.80, and 0.27.

## RESULTS

### Chemical Analyses of the Diets

The analyzed chemical compositions of the diets are presented in Table 2. Analyzed Ca of the diets was higher than formulated and consequently this raised the Ca:P ratio above anticipated (formulated 1.4:1 in PC and 1.5:1 in NC, analyzed as 1.5 to 1.7:1 in PC and 1.8:1 in NC). The phytase activity of the diets was greater than the expected levels, being <50, 715 to 744 and 1,880 to 1,960 FTU/kg in diets formulated to have 0, 500, and 1,500 FTU/kg, respectively, but still reflecting regular and super-doses.

**Table 2.** Calculated and analyzed nutrient and energy content of the experimental diets.

Description of diets	PC^1^	PC500	PC1,500	NC^2^	NC500	NC1,500
Phytase, FTU/kg	0	500	1,500	0	500	1,500
Calculated nutrients and energy:						
Protein, g/kg	230.5	230.5	230.5	230.3	230.3	230.3
ME, kcal/kg	3,020	3,020	3,020	2,941	2,941	2,941
Ca, g/kg	10.0	10.0	10.0	8.4	8.4	8.4
P, g/kg	6.9	6.9	6.9	5.5	5.5	5.5
Available P, g/kg	5.0	5.0	5.0	3.5	3.5	3.5
Ca:P	1.4	1.4	1.4	1.5	1.5	1.5
Na	1.8	1.8	1.8	1.7	1.7	1.7
K	7.9	7.9	7.9	7.9	7.9	7.9
Cl	2.2	2.2	2.2	2.2	2.2	2.2
Mg	1.5	1.5	1.5	1.5	1.5	1.5
Total amino acids, g/kg						
Lys	14.0	14.0	14.0	14.1	14.1	14.1
Met	5.0	5.0	5.0	5.0	5.0	5.0
Cys	4.0	4.0	4.0	4.0	4.0	4.0
Phe + Tyr	17.5	17.5	17.5	17.5	17.5	17.5
Analyzed nutrient content (g/kg):						
Ca	12.2	11.1	10.7	10.8	10.4	10.2
P	7.5	6.7	7.1	6.1	5.8	5.7
Ca:P	1.6	1.6	1.5	1.8	1.8	1.8
Phytate-P	1.7	2.4	2.2	1.7	2.0	1.8
Crude protein^3^	3.54	3.55	3.45	3.44	3.58	3.66
IP6 (mg/kg)	11.54	11.53	11.56	11.07	11.14	11.93
IP5 (mg/kg)	0.558	0.574	0.595	0.629	0.665	0.661
Na	1.8	1.7	1.5	1.7	1.6	1.6
K	9.0	8.5	9.7	9.0	8.9	8.5
Mg	1.4	1.3	1.5	1.4	1.4	1.3
Phytase^4,5,6^ (FTU/kg)	<50	715	1,880	<50	744	1,960

^1^PC: Positive control.

^2^NC: Negative control.

^3^Crude protein: Nitrogen analyses × 6.25.

^4^The enzyme used was Quantum Blue, provided by AB Vista, with an initial activity level of 5,000 FTU/kg. The premix was made to 150 FTU/kg and added at the rate of 10 g/kg or 30 g/kg to give activity levels of 500 or 1,500 FTU/kg, respectively.

^5^Dietary phytase quantification determined using ELISA method by AB Vista on final diets.

^6^Enzyme premix quantified at 56 FTU/kg using Quantum method by AB Vista.

### Growth Performance

Day 21 growth performance data are presented in Table 3. There were no treatment effects on initial body weight (**IBW**); therefore, this was not required to be added as a co-factor in the statistical model. There was aP × phytase interaction (*P* < 0.01) on BWG with phytase increasing BWG in birds fed the NC (quadratic, *P* < 0.001) and PC (quadratic, *P* < 0.01). Feed conversion ratio was improved (quadratic, *P* < 0.01) with the addition of phytase. There were no treatment effects on FI or mortality.

**Table 3.** Mortality, initial body weight (IBW, d 0) and body weight gain (BWG, d 21), feed intake (FI) and feed conversion ration (FCR) of broilers fed diets adequate or deficient in aP and Ca, with zero, 500 or 1,500 FTU/kg phytase, for 21 days.

Diet		IBW, g	BWG, g	FI, g	FCR	Mortality^2^%
		Simple effect means				
Negative control (NC)		41.1	729	1184	1.624	3.1
NC + 500 FTU/kg		40.9	852	1215	1.428	1.6
NC + 1,500 FTU/kg		41.2	849	1192	1.407	1.6
Positive control (PC)		41.8	840	1242	1.487	4.7
PC + 500 FTU/kg		41.4	874	1201	1.377	1.6
PC + 1,500 FTU/kg		41.3	882	1256	1.426	3.1
SEM		0.35	14.2	31.8	0.040	2.1
		Main effect means				
aP, %	0.35	41.1	810	1197	1.486	2.1
	0.50	41.5	865	1233	1.430	3.1
SEM		0.20	8.2	18.4	0.023	1.2
Phytase^1^	0	41.4	7814	1213	1.555	3.9
	500	41.1	863	1208	1.402	1.6
	1,500	41.3	865	1224	1.417	2.3
SEM		0.25	10.0	22.5	0.028	1.5
		*P*-values for main effects				
aP, %		0.153	<0.001	0.174	*0.095*	0.702
Phytase		0.749	<0.001	0.879	<0.001	0.673
aP × phytase		0.725	0.007	0.391	0.167	0.899
		*P*-values for orthogonal contrasts				
Phytase linear			<0.001		0.006	
Phytase quadratic			<0.001		0.005	
PC linear			0.043			
NC linear			<0.001			
NC quadratic			<0.001			

^1^FTU/kg phytase.

^2^Statistical analysis of mortality % was performed following the application of 1/(sqrtX+0.1) to allow for analysis of zero mortality.

8 replications per diet.

### Nutrient Utilization

#### Ileal Nutrient Utilization.

There was significant aP × phytase interaction on ileal DM, N, and P (*P* < 0.05) digestibility (Table 4). The digestibility of DM, N, and P was increased with the addition of phytase in the NC diets (quadratic, *P* < 0.01), but there was no effect of phytase on DM, N, or P digestibility in birds fed the PC. The digestibility of Ca increased as the dietary aP was reduced (*P* < 0.001) but decreased with the addition of phytase (linear, *P* < 0.001).

**Table 4.** Ileal nutrient digestibility of broilers fed diets adequate or deficient in aP and Ca, with zero, 500, or 1,500 FTU/kg phytase, for 21 days.

		Ileal digestibility coefficients			
		DM	N	P	Ca
		Simple effect means			
Negative control (NC)		0.537	0.513	0.417	0.466
NC + 500 FTU/kg		0.626	0.623	0.524	0.464
NC + 1,500 FTU/kg		0.590	0.586	0.410	0.295
Positive control (PC)		0.583	0.589	0.431	0.397
PC + 500 FTU/kg		0.569	0.569	0.371	0.287
PC + 1,500 FTU/kg		0.572	0.562	0.418	0.242
SEM		0.012	0.018	0.032	0.033
		Main effect means			
aP, %	0.35	0.584	0.574	0.450	0.408
	0.50	0.574	0.573	0.407	0.309
SEM		0.007	0.010	0.019	0.019
Phytase^1^	0	0.560	0.551	0.424	0.431
	500	0.598	0.596	0.478	0.376
	1,500	0.581	0.574	0.414	0.269
SEM		0.009	0.013	0.023	0.033
		*P*-values for main effects			
aP, %		0.328	0.956	0.107	<0.001
Phytase		0.016	0.058	0.560	<0.001
aP × phytase		<0.001	0.003	0.022	0.131
		*P*-values for orthogonal contrasts			
Phytase linear					<0.001
Phytase quadratic					0.824
PC linear		0.598	0.311	0.972	
NC linear		0.032	0.036	0.504	
NC quadratic		<0.001	<0.001	0.010	

^1^FTU/kg phytase.

8 replications per diet.

N: Nitrogen.

P: Phosphorus.

Ca: Calcium.

#### Total Tract Nutrient Retention.

The total tract nutrient retention (**TTR)** data are presented in Table 5. There was significant aP × phytase interaction on the TTR of DM, N, and P (*P* < 0.05). The TTR of DM and N was increased (quadratic, *P* < 0.05) when phytase was added to the NC treatments but there was no effect of phytase addition on the TTR of DM or N in birds fed the PC. The TTR of P increased (quadratic, *P* < 0.05) in birds fed the NC and there was no effect of phytase dose on the TTR of P in birds fed the PC. The TTR of Ca was greater in birds fed the NC compared with birds fed the PC diets (*P* < 0.01). At the highest level of inclusion of phytase in the NC, retention reached or exceeded that of the PC for most nutrients.

**Table 5.** Total tract nutrient retention of broilers fed diets adequate or deficient in aP and Ca, with zero, 500, or 1,500 FTU/kg phytase, for 21 days.

		Total tract retention coefficients			
Diet		DM	N	P	Ca
		Simple effect means			
Negative control (NC)		0.702	0.626	0.617	0.511
NC + 500 FTU/kg		0.734	0.679	0.703	0.569
NC + 1,500 FTU/kg		0.737	0.698	0.678	0.536
Positive control (PC)		0.729	0.699	0.581	0.513
PC + 500 FTU/kg		0.717	0.678	0.534	0.456
PC + 1,500 FTU/kg		0.718	0.674	0.568	0.446
SEM		0.007	0.011	0.023	0.029
		Main effect means			
aP, %	0.35	0.724	0.668	0.666	0.539
	0.50	0.721	0.683	0.561	0.472
SEM		0.004	0.006	0.013	0.017
Phytase^1^	0	0.716	0.663	0.599	0.512
	500	0.725	0.678	0.618	0.512
	1,500	0.728	0.686	0.623	0.491
SEM		0.005	0.008	0.016	0.020
		*P*-values for main effects			
aP, %		0.554	0.092	<0.001	0.008
Phytase		0.181	0.114	0.558	0.704
aP × phytase		0.002	<0.001	0.025	0.125
		*P-*values for orthogonal contrasts			
PC linear		0.336	0.147	0.903	
PC quadratic		0.320	0.360	0.147	
NC linear		0.002	<0.001	0.155	
NC quadratic		0.025	0.046	0.030	

^1^FTU/kg phytase.

8 replications per diet.

N: Nitrogen.

P: Phosphorus.

Ca: Calcium.

#### Phytate Hydrolysis.

The treatment effects on IP content of the gizzard are shown in Table 6. IP3 and IP2 were not detected in the gizzard. There was an aP × phytase interaction on the gizzard content of IP4 (*P* < 0.01). Whereas the addition of phytase to the PC had no effect on IP4 content, there was a quadratic increase in IP4 content when phytase was added to the NC (*P* < 0.001). There was a quadratic decrease (*P* < 0.001) in IP6 and IP5 content as phytase dose increased, whereas there was a tendency for a quadratic increase (*P* = 0.070) in inositol content with phytase supplementation. The inositol content of the gizzard was greater in the NC than PC diets (*P* < 0.05) but there was no effect on aP on IP6 or IP5 content in the gizzard.

**Table 6.** Inositol phosphate ester content of the gizzard of broilers fed diets adequate or deficient in aP and Ca, with zero, 500, or 1,500 FTU/kg phytase.

		mg/g DM^2^			
Gizzard		IP6^1^	IP5^1^	IP4	Inositol
		Simple effect means			
Negative control (NC)		3.58	0.50	0.55	0.09
NC + 500 FTU/kg		0.59	0.11	0.94	0.12
NC + 1,500 FTU/kg		0.52	0.07	0.67	0.14
Positive control (PC)		3.15	0.47	0.72	0.08
PC + 500 FTU/kg		0.65	0.12	0.83	0.11
PC + 1,500 FTU/kg		0.42	0.04	0.78	0.11
SEM		0.11	0.02	0.04	0.008
		Main effect means			
aP, %	0.35	1.56	0.22	0.72	0.12
	0.50	1.41	0.21	0.78	0.10
SEM		0.06	0.01	0.03	0.005
Phytase^3^	0	3.37	0.48	0.63	0.09
	500	0.62	0.11	0.89	0.12
	1,500	0.47	0.05	0.73	0.13
SEM		0.08	0.01	0.03	0.006
		*P*-values for main effects			
aP, %		0.474	0.243	0.107	0.018
Phytase		<0.001	<0.001	<0.001	<0.001
aP × phytase		0.606	0.129	0.005	0.315
		*P*-values for orthogonal contrasts			
Phytase linear		<0.001	<0.001	0.322	<0.001
Phytase quadratic		<0.001	<0.001	<0.001	0.070
PC linear				0.488	
NC linear				0.374	
NC quadratic				<0.001	

^1^Log transformation of IP6 and IP5 prior to analysis.

^2^IP2 and IP3 not detected.

^3^FTU/kg.

8 replications per diet.

IP6-4: Inositol phosphate ester with 6- 4 bound phosphate molecules.

In the ileum (Table 7), there was no significant aP × phytase interaction on the IP content; however, both the aP and phytase levels had a significant main effect. There was aP × phytase interaction on the disappearance of IP6 at the ileum (*P* < 0.01). Disappearance of IP6 increased quadratically in both the PC and NC diets (*P* < 0.001) and was numerically comparable between the PC and NC with 1,500 FTU/kg, but greater in the NC + 500 FTU/kg than when the same phytase dose was added to the PC diet (95% disappearance vs. 90%). In the absence of phytase, phytate disappearance was greater in the NC than PC.

**Table 7.** Inositol phosphate ester content of the ileum of broilers fed diets adequate or deficient in aP and Ca, with zero, 500, or 1,500 FTU/kg phytase, for 21 days.

			mg/g DM				
Ileum		IP6^1,2^ disappearance	IP6^1^	IP5^1^	IP4	IP3	Inositol
		Simple effect means					
Negative control (NC)		0.788	8.4	1.89	1.61	0.62	1.72
NC + 500 FTU/kg		0.938	3.6	0.55	1.04	0.67	2.26
NC + 1,500 FTU/kg		0.951	2.4	0.41	1.31	0.49	2.86
Positive control (PC)		0.671	14.9	2.29	1.69	0.44	1.23
PC + 500 FTU/kg		0.902	5.9	0.98	1.72	0.58	1.90
PC + 1,500 FTU/kg		0.953	2.5	0.45	1.48	0.63	2.07
SEM		0.0177	0.930	0.16	0.178	0.121	0.135
		Main effect means					
aP, %	0.35	0.892	4.79	0.95	1.32	0.59	2.28
	0.50	0.842	7.79	1.24	1.63	0.55	1.73
SEM		0.0102	0.537	0.094	0.126	0.070	0.078
Phytase^3^	0	0.730	11.67	2.09	1.65	0.53	1.47
	500	0.920	4.77	0.77	1.38	0.62	2.08
	1,500	0.952	2.44	0.43	1.39	0.56	2.47
SEM		0.0125	0.658	0.12	0.126	0.086	0.095
			*P*-values for main effects				
aP, %		0.001	0.008	0.035	0.041	0.670	<0.001
Phytase		<0.001	<0.001	<0.001	0.257	0.734	<0.001
aP × phytase		0.003	0.105	0.106	0.199	0.410	0.276
			*P*-values for orthogonal contrasts				
Phytase linear			<0.001	<0.001			<0.001
Phytase quadratic			0.013	<0.001			0.102
PC linear		<0.001					
PC quadratic		<0.001					
NC linear		<0.001					
NC quadratic		<0.001					

^1^Log transformation prior to analysis.

^2^Coefficients of IP6 disappearance

^3^FTU/kg.

No IP2 detected.

8 replications per diet.

IP6-3: Inositol phosphate ester with 6-3 bound phosphate molecules.

There was a decrease in IP6 and IP5 (quadratic, *P* < 0.05) and increase in inositol (*P* < 0.001) content in the ileum as phytase dose increased. IP6 (*P* < 0.01), IP5, and IP4 (*P* < 0.05) were lower, and inositol (*P* < 0.001) higher in the ileum of birds fed the NC compared with birds fed the PC. There was no effect of diet on IP3, and phytase dose had no effect on IP4 content in the ileum.

There was significant aP × phytase interaction on excreta IP5, IP4, IP3, and inositol content (Table 8). IP5 content in the excreta was reduced quadratically in birds fed the PC diet and in birds fed the NC (*P* < 0.05). However, in birds fed the NC diets, 500 FTU/kg had increased excreta IP5 to a greater extent than birds fed the PC (13% compared with 1%, respectively) and 1,500 FTU/kg decreased IP5 to a greater extent in the excreta of birds fed the PC compared with birds fed the NC (43% compared with 24%, respectively). The content of IP4 increased with the addition of phytase in the NC (quadratic, *P* < 0.001) and PC (linear, *P* < 0.001), as did IP3 in both the PC (quadratic, *P* < 0.001) and NC (linear, *P* < 0.001). The inositol content was higher in the NC than PC, and did not increase significantly in the NC with phytase supplementation, however, in the PC the increase was linear (*P* < 0.001). The IP6 content was influenced by phytase (*P* < 0.01), tending to decrease as the level of supplementation increased (quadratic, P = 0.055).

**Table 8.** Inositol phosphate ester content of the excreta of broilers fed diets adequate or deficient in aP and Ca, with zero, 500, or 1,500 FTU/kg phytase, for 21 days.

		mg/g DM				
Total tract		IP6^1^	IP5	IP4^1^	IP3	Inositol
		Simple effect means				
Negative control (NC)		35.6	3.60	1.83	0.17	1.21
NC + 500 FTU/kg		20.4	4.14	4.18	0.22	1.41
NC + 1,500 FTU/kg		12.9	3.14	7.05	0.24	1.46
Positive control (PC)		39.5	4.70	3.04	0.24	0.37
PC + 500 FTU/kg		20.7	4.76	4.93	0.18	0.89
PC + 1,500 FTU/kg		8.8	2.73	7.30	0.26	1.32
SEM		1.687	0.173	0.389	0.010	0.130
		Main effect means				
aP, %	0.35	22.92	3.63	4.35	0.21	1.36
	0.50	23.01	4.07	5.09	0.23	0.86
SEM		0.974	0.100	0.225	0.006	0.075
Phytase^2^	0	37.55	4.15	2.43	0.21	0.79
	500	20.55	4.45	4.55	0.20	1.15
	1,500	10.81	2.94	7.17	0.25	1.39
SEM		1.193	0.12	0.275	0.007	0.092
		*P*-values for main effects				
aP, %		0.715	0.004	<0.001	0.033	<0.001
Phytase		<0.001	<0.001	<0.001	<0.001	<0.001
aP × phytase		0.162	<0.001	0.018	<0.001	0.034
		*P-*values for orthogonal contrasts				
Phytase quadratic		0.055	<0.001	<0.001	0.067	0.357
PC linear			<0.001	<0.001	0.077	<0.001
PC quadratic			0.002	0.060	<0.001	0.212
NC linear			0.016	<0.001	<0.001	0.231
NC quadratic			0.003	<0.001	0.110	0.485

^1^Log transformation of IP4 and IP6 prior to analysis.

^2^FTU/kg.

8 replications per diet.

IP6-3: Inositol phosphate ester with 6- 3 bound phosphate molecules.

## DISCUSSION

### Growth Performance

There are many reports that the addition of phytase has the benefit of improving growth performance, in particular BWG and FCR, with the greatest improvements seen in P deficient diets (Sebastian et al., 1996; Liu et al., 2008; Akyurek et al., 2011; Cowieson et al., 2011). This study shows that phytase supplementation in diets low in available P is able to prevent the P deficiency symptoms and associated depression in growth performance, allowing the birds to match the growth performance of those birds fed Ca and P adequate diets. Overall, the specific benefits of phytase supplementation to nutrient deficient diets (NC) may vary between studies, according to the level of aP (and other nutrient) deficiency, ultimately giving different responses to phytase and the dose effects.

For BWG in the current study, the dietary level of aP had an important influence on the efficacy of phytase. The additional release of nutrients following phytate hydrolysis may lead to further interactions between nutrients and phytate within the digesta, and reduce the efficacy of phytase and potential for improvements in growth performance. In the NC diets where aP was limiting, subsequent Ca release from phytate hydrolysis can have detrimental effects on performance (Shafey, 1993) and may influence the Ca:aP ratio, which is important for phytase efficacy and performance (Shafey et al., 1990; Rao et al., 2006; Cowieson et al., 2011). The Ca:aP ratio is deemed more critical than absolute Ca and aP levels, with higher ratios having a detrimental effect on phytase efficacy (Shafey et al., 1990; Cowieson et al., 2011). Qian et al. (1997) reported that a greater Ca:aP ratio resulted in a reduction in growth performance, with the negative influence of a wide ratio being more apparent at the lower levels of phytase supplementation. Olukosi and Fru (2014b) reported that a wide Ca:aP ratio had a muting effect on the efficacy of phytase on performance parameters. The authors also reported that a wide Ca:aP ratio was more detrimental in reduced Ca and aP diets. In the current study, the analyzed dietary Ca content was higher than formulated, which, following the release of Ca from phytate hydrolysis, may have had the effect of further widening the Ca:aP ratio, leading to the observed difference in phytase effects between nutrient adequate PC and NC diets that are marginally deficient in aP (and often Ca).

### Nutrient Utilization

Ravindran et al. (2006) reported a phytase mediated increase in Ca digestibility, regardless of the dietary phytate content. In the current study, ileal Ca digestibility also was influenced by phytase dose and not dietary aP level, whereas Ca digestibility decreased with 1,500 FTU/kg phytase. This effect was likely a consequence of increased Ca released into the digesta following phytate hydrolysis. As well as potentially influencing the gut pH, absorption may already be saturated due to the higher than anticipated dietary Ca levels and this is confirmed by the lower Ca retention in birds fed the PC compared with birds fed the NC with reduced dietary aP and Ca levels. High Ca concentrations following release from phytate hydrolysis and thus a widening of the Ca:aP ratio can reduce the availability of other nutrients, primarily divalent minerals, in the digesta for absorption and increase their excretion (Shafey, 1993). The ratio between aP and Ca is important for nutrient utilization. Shafey et al. (1990) reported that the retention of P was not influenced over a wide range of dietary aP concentrations, provided adequate Ca was also provided to maintain the balance. However, when dietary Ca levels are high, P availability is observed to decrease (Tamim and Angel, 2003). High dietary Ca levels also have been reported to form insoluble Ca-phosphate complexes, which subsequently reduces the absorption of both Ca and P (Underwood, 1999).

Nutrient release following phytate hydrolysis can result in antagonism between the minerals in the digesta, reducing or preventing their absorption (Sebastian et al., 1996), with nutrient solubility and absorption being dependent on gut pH. Wilkinson et al. (2011) reported an inverse relationship between dietary Ca levels and P, Mg, and K digestibility. However in the current study we observed a positive correlation between Ca and P digestibility (r^2^ = +0.68, *P* < 0.001). Sebastian et al. (1996) observed an increase in TTR of P and Ca when phytase was added to low P diets, and Olukosi and Fru (2014a) reported an increase in Ca and P TTR with the addition of phytase to diets with a 2:1 Ca:aP ratio, but not when the ratio was increased to 2.5:1. This study by Olukosi and Fru (2014a) highlights the importance of the Ca:aP ratio in addition to consideration of the overall dietary Ca and aP concentrations as the authors also concluded that the negative influence of a wide Ca:aP ratio was more pronounced in low aP and Ca diets. In the current study, increasing the aP level reduced Ca and P TTR with a tendency for an increase in phytase dose to decrease P TTR (quadratic response). These results may indicate the levels of Ca in the NC, particularly with phytase supplementation, and also in the PC may have been much greater than the birds’ requirement, resulting in excretion of both Ca and P.

High doses of phytase are able to improve the digestibility of protein and P when phytate levels are low, as reported by Ravindran et al. (2006). Although in the current study the overall dietary phytate was comparable between treatments, by the time the digesta reached the ileum, there were differences in the IP6 content. Consequently, this may have then influenced the digesta environment, ileal nutrient utilization, and TTR due to its potential interaction and chelation with other nutrients in the digesta. Phytase increased N digestibility in the NC diets but not the PC, with digestibility being greater than the NC when 500 FTU/kg phytase was added, but not with 1,500 FTU. This difference in phytase dose response compared to a study by Ravindran et al. (2006) may be due to the additional release of Ca and P with 1,500 FTU/kg, influencing the Ca: aP ratio, thus changing the gut conditions (e.g., pH) for phytase action and nutrient absorption. The additional Ca present in the PC diets (further increased by phytate hydrolysis) may have bound to the phytate, preventing the hydrolysis of phytate and release of bound N. Similarly, the greater Ca content may have influenced the digesta pH such that the protein/N did not precipitate from the phytate-protein complexes in the digesta for its utilisation.

### Phytate Hydrolysis

As digesta flowed from the ileum through the remainder of the digestive tract, it appears that dietary aP had more of an influence on the phytate and the IP esters than it did in the gizzard. Li et al. (2016) reported that the proventriculus and gizzard are the most active sites of phytate hydrolysis by exogenous phytase and may be associated with the most soluble location for IP6 in the GIT, which is important for its interaction with phytase as suggested by Zeller et al. (2015). Inositol phosphate esters must be soluble in the digesta to be able to interact with phytase. Although IP3, IP2, and IP1 were below the limits of detection in the gizzard, this does not mean that they were not present, and will have some contribution to the overall IP content in the gizzard. It may be that IP2 was below the limits of detection, or may have contributed to the IP1/ inositol values. The decrease in IP6 and IP5 and increase in inositol in the gizzard in the presence of phytase reflects the hydrolytic action of the phytase occurring in the gizzard. Some of the IP3, IP2, and IP1 will be hydrolyzed and accounted for in the inositol content. It is unlikely that any of the esters will have been absorbed in the gizzard; however, due to the solubility of the lower esters, during the process of the removal of water from the gizzard to allow its function, these water-soluble lower esters may have passed out of the gizzard and were thus not detected on analysis. The relatively high IP6 content of the ileal digesta in the diets without phytase shows that little endogenous phytate hydrolysis had occurred up to this point. The phytate disappearance data show almost 10% greater ileal IP6 disappearance in the NC than PC diets. The higher ileal IP6 content in the PC than the NC (both without phytase) suggests that some endogenous phytase activity was occurring, but that in the PC diet there may have been phytate-interaction with other nutrients (i.e., minerals such as Ca), preventing its binding with endogenous phytase for hydrolysis.

It appears that the majority of the dietary IP6 may be hydrolyzed (step-wise) through to IP4 and IP3, with the IP5 and IP4 esters produced by phytase hydrolysis being more susceptible to hydrolysis than the IP5 and IP4 esters occurring in the feed. Li et al. (2016) reported the accumulation of IP6 in the distal ileum regardless of dietary factors, suggesting that in the absence of phytase, there are few dietary influences on the disappearance of IP6. When Li et al. (2016) supplemented phytase, there were no observed differences in the ability of phytase to hydrolyse phytate at different levels of dietary Ca, aP, and phytate-P; however, the low dietary Ca levels used were generally higher than the “high dietary Ca” treatments in many other studies. Ca is regularly reported as having a negative effect on phytase activity when present at high levels, with phytate-P hydrolysis reported to decrease with increasing dietary Ca (Tamim and Angel, 2003; Tamim et al., 2004). In the study reported by Li et al. (2016) the chelation sites for IP6 with Ca may have already been full at the “low” Ca level, as a result of which no differences would have been observed by increasing the Ca levels further, either through dietary manipulation or following phytate hydrolysis. As there were treatment effects in the current study on the excreta content of IP5, IP4, and IP3, but not in the ileum (with numerically greater concentrations in the excreta than the ileum, even in the treatments in which phytase was absent), some IP6 hydrolysis appears to have occurred in the cecum. Phytate hydrolysis in the cecum may be a result of bacterial action, as the phytase enzyme would be expected to be completely degraded by this point (Zeller et al., 2015). Perhaps the greater IP4 content of the phytase supplemented diets may reflect the inability of the cecal bacteria to hydrolyze exogenous phytase-produced IP4, which may have a different structure to the IP4 present in the feed (M. R. Bedford, AB Vista, Marlborough, UK, personal communication). Zeller et al. (2015) reported different enantiomers of the IPs produced during phytate hydrolysis.

Additional observation of the ratios between IP6 and IP4 contents of the ileal digesta and excreta suggests this may be the case, as highlighted by the identification of a negative correlation between excreta IP6 and IP4 content. In the ileum and excreta, the ratio of IP6:IP4 decreased as phytase increased, in both the PC and NC diets, reaching 1:1 in the excreta when high doses of phytase had been used. In the absence of phytase in the ileum, the IP6 content was greater than the IP4. Strong negative correlations between excreta IP4 and IP6 content were identified (r^2^ = −0.82; *P* < 0.001). As the phytase dose increased, the ratio between IP6 and IP4 decreased as IP6 was hydrolyzed and IP4 content marginally increased. Differences were more apparent in the excreta, where the ratio decreased as phytase dose increased. Hydrolysis of IP6 led to increased IP4 content, which without the addition of the super-dose of 1,500 FTU/kg phytase would have accumulated in the digesta. Instead, the IP6:IP4 ratio came closer to 1:1 as IP4 was cleared by hydrolysis. These results suggest that IP4 is more susceptible to hydrolysis post ileum in the control diets than the phytase supplemented diets. This may be explained by a difference in structure between the IP4 produced by endogenous and exogenous phytase sources and the ability of bacteria in the ceca to hydrolyze these molecules. However, the use of super-doses of phytase is more likely to hydrolyze the IP4 to the lower esters, as indicated by the IP6:IP4 ratio decreasing to 1:1 with 1,500 FTU/kg phytase.

The interactive effect between dietary aP and phytase appeared to become irrelevant for the inositol content in the excreta. As expected, inositol content increased with increasing doses of phytase, indicating that high levels of phytase are more effective at increasing inositol concentrations than regular levels. Inositol content of the excreta is numerically lower than the content in the ileum, which suggests potential uptake of inositol in the terminal region of the ileum, or by bacteria residing in the ceca.

## CONCLUSION

Phytase supplementation was able to prevent the depressions in growth performance observed in the NC diet without phytase, being comparable to the PC, with or without phytase. There was no interaction on phytate hydrolysis in the ileum, with phytase decreasing IP6 and increasing inositol contents. Nutrient imbalance may be mediated by nutrient release from phytate hydrolysis and have further downstream effects on hydrolysis of the inositol phosphate esters and gut characteristics.
